# Dual roles of nitric oxide in the regulation of tumor cell response and resistance to photodynamic therapy

**DOI:** 10.1016/j.redox.2015.07.015

**Published:** 2015-07-31

**Authors:** Valentina Rapozzi, Emilia Della Pietra, Benjamin Bonavida

**Affiliations:** a Department of Medical and Biological Sciences, University of Udine, P.le Kolbe 4, 33100 Udine, Italy; b Department of Microbiology, Immunology and Molecular Genetics, University of California Los Angeles, Los Angeles, CA 90095, USA

**Keywords:** ABC, ATP-binding cassette, ABCG2, ATP-binding cassette sub-family G member 2, AIF, apoptosis inducing factor, ALA, aminolevulinic acid, BCC, basal cell carcinoma, BCG, Bacillus Calmette-Guerin, CG, cholangiocarcinoma, CTL, cytotoxic T-lymphocyte, DR4/DR5, TRAIL death receptors, EGF, epithelial growth factor, EMT, epithelial mesenchymal transition, FASL, fas ligand, FDA, food and drug administration, 5-FU, 5-fluorouracil, GI, gastrointestinal, GSNO, S-nitrosoglutathione, HBD, hematoporphyrine-derivative, iNOS, inducible nitric oxide synthase, L-NAME, l-N^G^-Nitroarginine methyl ester, MAL, methylaminolevulinate, MDR, multidrug resistance, mPEG, monomethoxy-polyethylene glycol, NF-kB, nuclear factor kappa-light-chain-enhancer of activated B cells, NK, natural killer, ^3^O_2_, molecular singlet oxygen, ^1^O_2_, singlet oxygen, PARP, poly ADP ribose polymerase, Pba, pheophorbide a, PDT, photodynamic therapy, PS, photosensitizer, RIPT-1, receptor activity protein I, RKIP, Raf kinase inhibitor protein, ROS, reactive oxygen species, Ru (NO)(NO)(ONO)(pc), nitrosyl-phtalocyanin ruthenium complex, SCC, squamous cell carcinoma, SNAP, S-nitroso-N-acetylpenicillamine, SOD, superoxide dismutase, TNF-α, tumor necrosis factor alpha, TRAIL, TNF-related apoptosis-inducing ligand, TNF-R1/R2, tumor necrosis factor receptor 1/receptor 2, UV, ultraviolet, YY1, Yin Yang 1, Nitric oxide, Photodynamic therapy, Tumor response, Resistance, Molecular pathways.

## Abstract

Photodynamic therapy (PDT) against cancer has gained attention due to the successful outcome in some cancers, particularly those on the skin. However, there have been limitations to PDT applications in deep cancers and, occasionally, PDT treatment resulted in tumor recurrence. A better understanding of the underlying molecular mechanisms of PDT-induced cytotoxicity and cytoprotection should facilitate the development of better approaches to inhibit the cytoprotective effects and also augment PDT-mediated cytotoxicity. PDT treatment results in the induction of iNOS/NO in both the tumor and the microenvironment. The role of NO in cytotoxicity and cytoprotection was examined. The findings revealed that NO mediates its effects by interfering with a dysregulated pro-survival/anti-apoptotic NF-κB/Snail/YY1/RKIP loop which is often expressed in cancer cells. The cytoprotective effect of PDT-induced NO was the result of low levels of NO that activates the pro-survival/anti-apoptotic NF-κB, Snail, and YY1 and inhibits the anti-survival/pro-apoptotic and metastasis suppressor RKIP. In contrast, PDT-induced high levels of NO result in the inhibition of NF-kB, Snail, and YY1 and the induction of RKIP, all of which result in significant anti-tumor cytotoxicity. The direct role of PDT-induced NO effects was corroborated by the use of the NO inhibitor, l-NAME, which reversed the PDT-mediated cytotoxic and cytoprotective effects. In addition, the combination of the NO donor, DETANONOate, and PDT potentiated the PDT-mediated cytotoxic effects. These findings revealed a new mechanism of PDT-induced NO effects and suggested the potential therapeutic application of the combination of NO donors/iNOS inducers and PDT in the treatment of various cancers. In addition, the study suggested that the combination of PDT with subtoxic cytotoxic drugs will result in significant synergy since NO has been shown to be a significant chemo-immunosensitizing agent to apoptosis.

## Highlights

• PDT-mediated cytotoxic and cytoprotective effects depend also by the induction of NO from tumor.
• The PDT-induced NO modulates the dysregulated NF-kB/Snail/RKIP loop.
• The direct role of NO induction by PDT was corroborated by the use of the NO inhibitor, l-NAME.
• The combination of an NO donor and PDT resulted in a increased cytotoxic effect, *in vitro* and *in vivo*.
• Novel potential therapeutic applications are proposed for the use of PDT combined with NO donors.

## Graphical abstract

**Figure:**
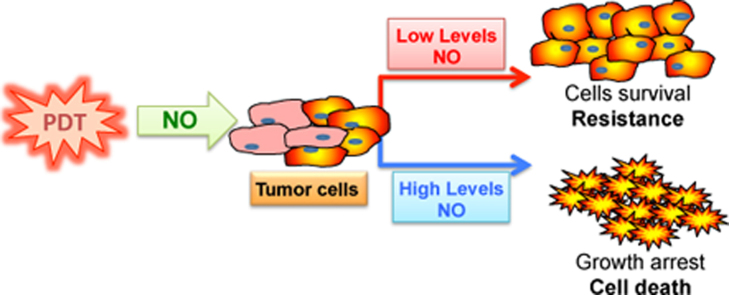

## 1 Introduction

Photodynamic therapy (PDT) is a therapeutic modality for certain diseases including cancer. PDT consists primarily of a photosensitizer (PS) and followed by light irradiation of a predetermined wavelength [1]. However, oxygen is an essential mediator of PDT [1,2]. The PDT-generated reactive oxygen species (ROS) and singlet oxygen (^1^O_2_) cause damage to the tumor tissues and cells by inducing necrosis and apoptosis. Optimally, the selective effect of PDT is through the localization of the photosensitizer in the desired region and the precise delivery of the light source to the treated areas. The PDT activity has its own limitations, for example, its effect on metastatic cancer lesions.

### 1.1 The photosensitizer (PS)

Most of the photosensitizers (PSs) used in cancer therapy belong to the protoporphyrin family and are based on a tetrapyrrole structure. An ideal sensitizer must have an absorption peak between 600 and 800 nm (red to deep red). High wavelengths greater than 800 nm produce a limited source of photons since they are poor in exciting oxygen to its singlet state and, thus, reduce reactive oxygen species that are required for cytotoxic effects. The mechanism of tumor localization of PS has been investigated revealing the role of the leaky blood vasculature in cancers and the absence of drainage by the lymphatic system leading to retention [3]. Also, some PSs bind to low density lipoproteins and bind cancers overexpressing LDL receptors and, thus, are more directed on tumors [4]. Other reports also demonstrated the use of PSs covalently linked to binding agents directed at cancer bearing receptors on the tumor cell surface [5]. Such coupling agents include antibody molecules, antibody fragments, peptides, proteins, EGF, etc.

### 1.2 Light sources for radiation

Red and infrared radiation penetrate into tissues more deep and only in the range of 600 to 800 nm to generate singlet oxygen for toxicity [6]. The choice of light source is dependent on the PS used and is based on the PS absorption, the disease and its size. The fluence rate affects significantly the PDT response [7]. Both lasers and incandescent light sources have been used for PDT and result in similar effects [8]. More detailed analyses of light sources have been reviewed elsewhere [9–14].

### 1.3 Photochemistry

The light exposure on the PS undergoes a shift from the ground (singlet) state to an excited singlet state. The latter undergoes crossing to an excited triplet state and this can result in the formation of radicals (ROS) (Type I reactions) or transfer the energy to molecular singlet oxygen (^3^O_2_) to form singlet oxygen (^1^O_2_) (Type II reactions). Singlet oxygen is the predominant cytotoxic molecule in PDT [9].

## 2 Dual cytotoxic and cytoprotective roles of PDT

### 2.1 PDT-mediated cytotoxicity

Various PSs target different organelles and subcellular compartments and mediate cytotoxic effects, which will vary based on the targeting – and the sensitivity of the tumor cells to cytotoxic damage [13,15]. Three major types of cell death by PDT have been reported, namely, (1) apoptosis, (2) necrosis and (3) autophagy. Apoptosis is the major cell death mechanism induced by PDT [9,14].

### 2.2 PDT-mediated cytoprotection

Many cancer cells are not sensitive to PDT-mediated cytotoxicity. Tumor cells develop various mechanisms to protect them from cell death-induced by PDT and many other cytotoxic agents. For instance, certain cancer cells have high levels of antioxidants [16]. Others have overexpression of detoxifying enzymes for ROS [17] and may have protective genes induced by PDT and/or overexpress several anti-apoptotic gene products [18–20]. A more detailed analysis on the mechanisms discussed above would be reported below.

## 3 Clinical applications of PDT in a variety of human cancers

Historically, Dougherty et al. [21] reported the first clinical study of the application of PDT in patients with a variety of malignant diseases. They treated the patients with PDT with a hematoporphyrine-derivative (HBD). They achieved complete and partial responses in 111 out of 113 treated cancer patients. These initial successful findings of the application of PDT in cancer was followed by hundreds of clinical trials [9,22,23]. PDT was most effective on the surface of lesions due to the limited penetration of the light source deep into the tissues; the range of tumor destruction did not overall exceed one centimeter. Briefly, a few examples of the therapeutic applications of PDT in various cancers are presented.

Còrdoba et al. [24] and Nestor et al. [25] reviewed the response of PDT treatment in premalignant and malignant skin tumors. Noteworthy, PDT was approved in the USA, Canada and Europe for its use in actinic keratosis and also in the European Union and Canada for basal cell carcinoma (BCC). In actinic keratosis, randomized controlled trials reported complete response rates (82–100%) for PDT with aminolevulinic acid (ALA-PDT) or methylaminolevulinate (MAL-PDT) as compared to 67 to 100% for cryotherapy and 74–94% for the application of 5-FU cream at 12 and 24 months [26,27]. In BCC, PDT was superior to cryosurgery or surgery for a selected subset of patients. Also, PDT actinic is a superior cosmetic outcome compared to surgery [28,29]. The use of MAL-PDT was found to be a safe and effective treatment for BCC in patients with Gorlin's syndrome and its efficacy is correlated to the thickness of the region [30]. PDT was also found to have chemo-preventive activity in patients with the Gorlin's syndrome [31].

PDT has been employed in the treatment of head and neck cancer, successfully [32]. Of interest, the study evaluated PDT treatment of patients with advanced diseases and not responding to tumor treatments. They applied Foscan-mediated PDT in 128 patients with a single session of PDT. There was a remarkable response in tumor destruction and complete local tumor clearance [33]. These findings suggest that PDT may be an alternative treatment for patients with early head and neck tumors.

Tumors of the digestive system have been grouped into PDT of the esophagus [34] and tumors beyond the esophagus. The U.S. FDA approved photofrin-mediated PDT for patients with Barret's esophagus and high grade dysplasia who did not undergo surgery [34]. PDT has been applied to other GI digestival tumors under the stomach [35,36], cholangiocarcinoma (CG) [37], with a therapeutic response on unresectable pancreatic cancers [38], and on colon or rectal cancers [39,40].

Intraperitoneal (ovarian, gastrointestinal, sarcoma) have been treated with PDT [41]. There was a suggestion that the median survivals of two years for ovarian cancer and one year for gastrointestinal cancer have been beneficial by PDT compared to controls.

Several reports have shown that the results of PDT treatment of prostate cancer. These studies established the potential use of PDT in prostate cancer and toxicity was considered as a determining factor [42–44].

Superficial bladder cancer is a good target for PDT. Long-term desirable responses of 20–60 of patients who were treated and many of those patients had recurrent disease following BCG treatment [45,46]. While PDT treatment for bladder cancer has been approved in the EU and Canada, it is not yet approved by the U.S. FDA.

In non-small cell lung cancer, the results of PDT treatment are encouraging [47,48]. In patients with malignant pleural mesothelioma, a randomized phase III study compared PDT with surgery and the findings demonstrated the benefit of PDT over surgery [49].

Promising clinical findings of PDT in brain tumors were reported [50,51]. However, more phase III clinical trials are needed to place PDT as superior to other therapeutics in certain cancers. Also a number of applications of laser technologies for accurate dosimetry are needed.

PDT has also been used in the treatment of mycosis fungoides, an indolent subtype of cutaneous T-cell lymphoma. It was reported that consecutive PDT treatments are adjunct for treatments of mycosis fungoides with good cosmetic results [52].

## 4 Molecular mechanisms of PDT-mediated cytotoxicity and resistance

### 4.1 PDT-mediated cytotoxicity

It was stated that active PDT-mediated cytotoxicity resulted from apoptosis, necrosis and autophagy. Apoptosis is a mechanism of programmed cell death that is activated by external death ligands (type I) or intracellular effects on the mitochondria (type II) via chemotherapeutic drugs, antibodies, toxins, DNA damaging agents, etc. For example, type I apoptosis is activated from the binding of death ligands (CTL, NK), FasL, TNF-α, and TRAIL to corresponding receptors Fas, TNF-R1/R2, DR4/DR5, respectively. Sensitive cells are induced to apoptosis by activation of caspase-8 and the effector caspase-3 leading to activation downstream of PARP and DNA fragmentation. Type II apoptosis results from altering the mitochondria permeability membrane and inducing the release of cytochrome *c* and smac/DIABLO, which lead to the activation of caspase-9. Subsequently, there is activation of caspases 7 and 8 and caspase 3, a merging point of type I and type II, and leading downstream to apoptosis. In addition to cytochrome c and smac/DIABLO, AIF is also released and activates apoptosis by a caspase-independent mechanism. Most PDT induce type II apoptosis [14]. PDT also photooxidizes lysosomes leading to the rupture and release of cathepsins which induce Bid cleavage and permeabilization of the mitochondrial outer membrane [53]. Cell death induced by PDT by necrosis has been observed and the underlying mechanism is not really clear [54,55]; although it has been reported that the activation of the receptor activity protein I (RIPT-1), excessive ROS production, lysosomal damage, and calcium are involved [55,56].

PDT also induces autophagy, a lysosomal pathway involved in the degradation and recycling of intracellular proteins and organelles. Autophagy can be induced by oxidative stress [57,58].

### 4.2 Resistance to PDT-mediated cytotoxicity

Tumor cells utilize several mechanisms to be resistant to the cytotoxic effect of PDT [59,60]. Briefly below, we discuss the most pertinent of those mechanisms, and several of those have been revealed through the utilization of PDT-resistant tumor cell lines. The mitochondrion plays an important role and any perturbation of the content enzymes in cancer cells may result in PDT resistance since PSs mediate their activity in the mitochondria [61,62].

Tumors resistant to several chemotherapeutic drugs exhibit the MDR phenotype [63–65]. The findings of the role of the MDR (Pgp) and the resistance to PDT are controversial depending on the cell lines, the kind of the PS used, and how the PS interacts with the MDR proteins. Another ABC transporter capable of inducing drug resistance in breast cancer cells was termed ABCG2 [66,67]. There is a correlation between the expression of ABCG2 and the resistance to PDT as a function of the PS structure [68]. Overall, high Pgp and ABCG2 expressions potentiate resistance to PDT and inhibitors of those transporters may reverse resistance [69].

DNA damage may be induced by PDT [70]. PDT induces activation of early response genes [61] with the activation of cell survival pathways. Hence, the hyper activation of survival pathway may result in resistance of PDT-mediated cytotoxicity. In addition, tumor cells overexpress anti-apoptotic gene products that play a role in the resistance of PDT-mediated cell death.

PDT is also antagonized by antioxidant defense mechanisms including the glutathione system, superoxide dismutase (SOD), catalase, and lipoamine dehydrogenases [17,71]. The increase of heat shock proteins may also be involved in PDT resistance [72]. Modification of the extra cellular matrix in tumor cells affects PDT cell toxicity [59].

The role of NO in resistance has been controversial and depending on the level of NO. Low levels of NO mediate chemo and radio-resistance whereas high levels of NO mediate cytotoxicity and sensitize tumor cells to chemo-immunotherapy (see chapter Bonavida and Garbon in this volume). The subset of NO in PDT will be discussed below separately.

## 5 Dual roles of NO-mediated anti-tumor effects

The induction of NO in tumor cells may result in some protective effects by mediating cell proliferation, survival, and resistance. For example, reports by Sikora [73] reported that the inhibition of inducible nitric oxide synthase (iNOS) repressed the growth of human melanoma *in vivo* and synergized with cisplatin. Noteworthy, Eyler et al. [74] reported that glioblastoma stem cells expressed higher levels iNOS than normal stem cells and the iNOS inhibition reduced cell proliferation *in vitro. In vivo*, in a mouse xenograft model, an iNOS inhibitor slowed tumor progression and prolonged survival. Such studies and others demonstrated that many cancer cells utilized low levels of iNOS/NO to reduce apoptosis, to stimulate cell proliferation and to induce invasion and metastases. NO at low concentration can act as antioxidant [75] and also S-nitrolsylate proteins that activate pro-survival pathways [76].

The dual roles of NO have been the subject of many studies in both NO-mediated cytoprotective and cytotoxic effects. NO at high concentration, in the µM range, is cytotoxic due to its conversion to oxidized intermediates that damage the DNA, exerts lipid peroxidation on the membrane and inhibits certain proteins by S-nitrosylation [77–80]. NO dual contrasting roles in PDT have been reviewed elsewhere [81–84].

## 6 NO donors and PDT

Recently, several groups have begun to synthesize NO donors to promote PDT-mediated anti-tumor cytotoxicity. For instance, Carneiro et al. [85] reported the synthesis and activity of a nitrosyl-phtalocyanin ruthenium complex [Ru (NO) (NO) (ONO) (pc)] and studied its effect on a murine melanoma cell line, B16F10, in the presence or absence of light irradiation. Their findings demonstrated that the complex was more effective in inhibiting B16F10 cell growth than the free [Ru (pc)] demonstrating the importance of NO release. Also, the encapsulation of the complex into liposomes was >25% more effective than the non-capsulated complex. The phototoxicity of the complex on B16F10 cells was primarily due to apoptosis.

Giles et al. [86] designed 2 photolabile NO-releasing prodrugs, tert-butyl-S-nitrosothiol and tert-dodecaire-S-nitrosothiol. These prodrugs have better kinetics of NO release than available nitrosothiols and are stable *in vitro* in the absence of radiation. Experimentally, irradiation increased the cytotoxic activity of these prodrugs and the authors suggested their therapeutic potential. In subsequent studies, the same group designed a superior NO donor than the conventional GSNO SNAP [87]. Tert-dodecaire-S-nitrosothiol released high NO than GSNO or SNAP, and exhibited a photodynamic response. The authors concluded that this compound is the most effective known S-nitrosothiol for PDT application. Rapozzi et al. [88] reported the superior activity of a new complex DR2 constituted of a PS (Pheophorbide *a*, Pb*a*) connected to a non-steroidal antiandrogen molecule able to release cytotoxic NO under the exclusive control of light in prostate cancer cells.

Reported studies implicated the role of the high level of NO in its interference with the dysregulation of NF-κB/Snail/YY1/RKIP in cancer cells [89]. This loop provided the tool to examine its role and implication in PDT on one hand and its regulation in NO-mediated PDT treatment resulting in either the cytoprotective tumor recurrence or antitumor cytotoxicity. Below briefly, we present our findings that have been recently published [90,91].

### 6.1 NO-mediated PDT-induced cytotoxicity

It is well established that treatment with PDT results in the induction of NO through the activity of the PS and light as a result of the induction of iNOS [81,82]. The induction of NO by PDT is the result of both the activity of iNOS expression by both the tumor cells and the tumor microenvironment [83,84]. Whether the NO-mediated induction by PDT plays a major role in PDT-mediated cytotoxicity was investigated. This hypothesis was examined *in vitro* in a tumor cell model using the amelanotic murine cell line, B78-H1 [90].

In this model, Pb*a* was used as the PS. Treatment of tumor cells with Pb*a* induced iNOS expression and the level of iNOS are a function of the concentration of Pb*a* used. In addition, treatment with Pb*a* inhibited tumor cell viability as assessed by a reduction of metabolic activity of the treated cells. The direct role of NO-induction by PDT was corroborated by the use of l-NAME, an inhibitor of NO, and such a treatment reversed the cytotoxic activity.

We then examined the effect of Pb*a* on the expression of the loop gene products. Treatment of B78-H1 cells with Pb*a* resulted in the inhibition of NF-kappa B and Snail expressions while upregulating the expression of RKIP. In addition, treatment with Pb*a* induced the activation of caspases 3 and 7 above control levels and suggested that the cytotoxic mechanism involved apoptosis. The *in vivo* findings of Pb*a*-induced inhibition of cell proliferation and induced cytotoxicity were corroborated in a murine bearing B78-H1 tumor cells whereby the administration of mPEG Pb*a* resulted in significant inhibition of tumor growth *in vivo* [90].

The above findings demonstrated clearly that treatment of B78-H1 tumor cells with PDT resulted in cytotoxicity via inhibition of the constitutively activated NF-kappa B pathway, responsible for cell proliferation and viability, and downstream inhibition of its target gene product, Snail, and resulting in the derepression of the metastasis suppressor gene product RKIP. In addition, the induction of RKIP potentiated the inhibition of NF-kappa B activity as reported [92]. PDT-mediated effects are the result, in part, of the induction of NO. NO has been reported to inhibit the NF-κB activity via the S nitrosylation of p50 and p65 [93]. Thus, based on these findings, we have postulated that NO-mediated PDT cytotoxic activity may be enhanced by the combined treatment of PDT and an NO donor. Accordingly, we have used the NO donor, DETANONOate, which was reported to have a significant effect in interfering with the loop in cancer cells and resulting in the inhibition of cell survival and sensitization to chemotherapeutic drugs [89]. Therefore, the combination of PDT and DETANONOate was examined and the findings revealed, in contrast to single agent treatment alone, that the combination resulted in significant potentiation of (a) inhibition of metabolic activity (b) inhibition of NF-κB activity (c) inhibition of Snail and (d) upregulation of RKIP. In addition, *in vivo* studies in mice revealed that the combination of Pb*a* and DETANONOate resulted in significant inhibition of tumor growth compared to single treatment alone and significant prolongation of survival in mice [90].

**Fig. 1:**
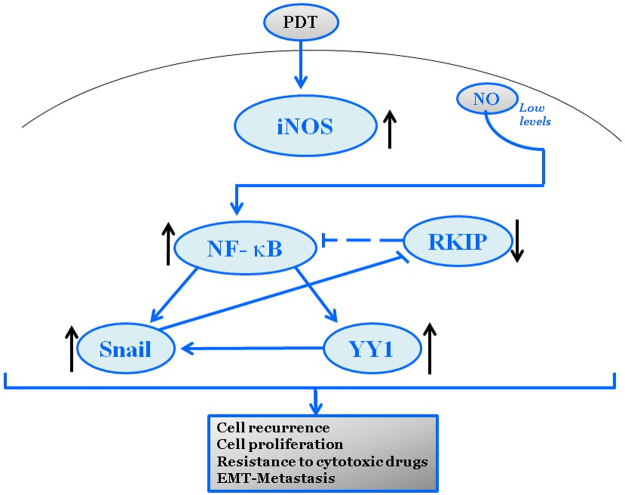
Cytoprotective role of PDT-induced NO.

Overall, the above findings demonstrated clearly that the NO-induced by PDT plays a pivotal role in PDT-induced cytoxicity and that the addition of exogenous NO potentiated the cytotoxic activity against the tumor cells. A schematic diagram representing PDT-induced NO-mediated cytotoxity is shown in Fig. 1.

### 6.2 NO-mediated PDT inhibition of cytotoxicity and epithelial mesenchymal transition

The protective role of NO-induced by PDT in cytoprotection has been the subject of many reports and recently reviewed by Girotti [82]. We have reported that the level of NO-induced by PDT has contrasting effects on the NF-κB/Snail/YY1/RKIP loop i.e. low level of NO activates NF-κB, YY1, and Snail and inhibits RKIP whereas high level inhibits NF-κB, Snail, YY1, and induces RKIP [90,91,94].

It was also reported that the activity of the above loop not only regulated cell survival and viability, but also regulates the epithelial to mesenchymal transition (EMT) [95]. Thus, based on the findings that a suboptimal PDT-treatment resulted in a transient inhibition of tumor cell growth followed by recurrence, we examined the role of PDT-induced NO in a model of tumor cell recurrence as well as its role in EMT [91]. The tumor model used consisted of the human PC3 prostate cancer cell line. A suboptimal concentration of Pb*a* was used, which had no effect on PC3 cells, and treatment was repeated 4 or 8 times and the properties of the treated tumor cells were examined at these time intervals. There was significant enhancement of cell proliferation and with a mild and constant production of iNOS in the cells. Noteworthy, there was significant activation of NF-κB and YY1 [91] expressions following 8 treatments along with inhibition of RKIP. Also, there was significant activation of the AKT pathway, which regulates NF-κB activity [91]. In addition, the modulation of the loop by PDT treatments resulted in the induction of the EMT phenotype of PC3 cells as determined by inhibition of E-cadherin and the induction of vimentin. The direct role of NO on both the effects on the loop and EMT by repeated PDT treatments was corroborated by the use of the NO-inhibitor, l-NAME, that resulted in the reversal of the observed effects.

**Fig. 2:**
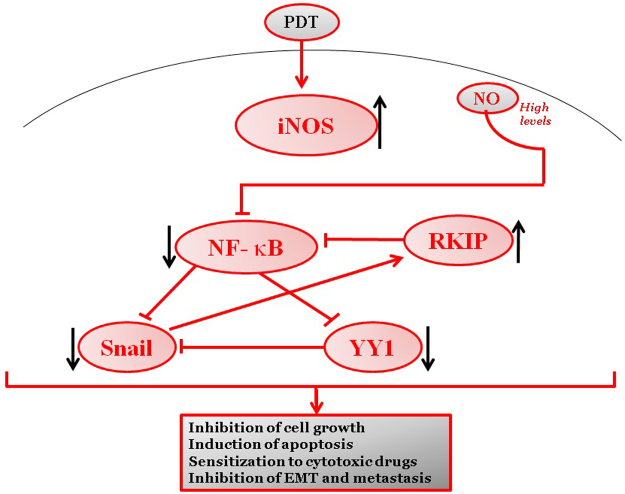
Cytoyoxic role of PDT-induced NO.

The above findings demonstrated a mechanism by which the cytoprotective effect of PDT is mediated by low levels of NO-induction resulting in cell proliferation and EMT [91]. A schematic diagram representing these effects is shown in Fig. 2.

We hypothesized that low levels of PDT-induced NO may activate the loop and result in cell proliferation and tumor recurrence. However, high levels of induced NO will inhibit the loop and result in the inhibition of cell proliferation, reduced cell viability, and induction of cell apoptosis.

## 7 Concluding remarks and future directions

### 7.1 Concluding Remarks

It is clear that the induction of NO by PDT plays an essential role in its cytotoxic anti-tumor activity provided the amount of NO released is optimal for mediating cytotoxicity. Since the amount of NO-induced by PDT varies and is dependent on the tumor tissue, the PS used, the light source, one may consider various means to overcome such limitations. The combined treatment of NO donors and PDT resulted in a significant synergistic cytotoxic activity using tumor model systems described here. Clearly, the overall activity mediated by PDT-induced NO or the addition of exogenous NO resulted, in part, in the interference of a dysregulated loop (the pro-survival/anti-apoptotic/NF-κB/Snail/YY1/RKIP) found to be present in many cancers. This dysregulated loop has been shown to be central for tumor cell survival, proliferation, resistance, invasion, angiogenesis, EMT and metastasis. Thus, the combined treatment of PDT and NO donors results in pleiotropic activities that not only induce cytotoxicity against a tumor, but also, prevent invasion and metastasis as well as reverse resistance. Several NO donors have been used and others are being developed for their anti-tumor mediated activities used alone or in combination with sensitizing agents and drugs. Clearly, the therapeutic implication for the use of the combination of NO donors and PDT in cancer patients warrants clinical trials to determine toxicity and efficacy. At present, there have been a few ongoing clinical trials with NO donors as single agents, but clearly, these would be followed with clinical trials for the combination treatments with PDT.

### 7.2 Future directions

Several future directions for investigations are being currently contemplated, for example, analysis of the effect of the combination of NO donors and PDT on cytotoxicity on cancer stem cells. Also, several reports have demonstrated the significant chemo and immuno-sensitizing activities of NO donors. Thus, analysis of the combination of NO donors and suboptimal PDT in resistance as chemoimmunosensitizing agents are warranted for investigation. There are also reports on the analysis of the superiority of NO-drug conjugates in comparison with single agents. Novel synthesized PDT-NO complexes have been synthesized and are currently being investigated. Preliminary findings demonstrated that a selected PDT-NO complex is more cytotoxic than single agents alone or combination [88]. In addition, the application of nanoparticles coated with the PS-NO complex will be examined for their superior activity. The findings demonstrating PDT-induced NO on the dysregulated NF-κB/Snail/YY1//RKIP loop suggested that inhibitors of NF-κB/Snail/YY1 or inducers of RKIP may be useful if used in combination with PDT for anti-tumor cell activity, and such studies are currently being explored.

This schematic diagram represents treatments with suboptimal PDT result in the low induction of iNOS and resulting in low levels of NO. Under these conditions, NO induces the expressions and activities osf NF-κB and downstream its target gene products Snail and YY1. The overexpression of SNAIL represses the transcription of RKIP. Thus, under the conditions of low levels of NO, the modified dysregulated loop results in the overexpression of NF-κB, YY1, and Snail and the inhibition of RKIP expression. Hence, the tumor cells undergo recurrence, cell proliferation, resistance to cytotoxic drugs, expression of the EMT phenotype, and metastasis.

This schematic diagram represents optimal PDT treatments which result in the high induction of iNOs expression and high levels of NO. High levels of NO inhibit NF-κB, Snail, and YY1 activities and expressions and resulting in the derepression and overexpression of RKIP. In addition, the overexpression of RKIP, in turn, potentiates the inhibition of NF-κB and its target genes Snail and YY1. These manifestations by high levels of NO result in inhibition of tumor cell growth, induction of apoptosis in sensitive cells, sensitization to cytotoxic drugs, and inhibition of EMT and metastasis.
